# Influence of Mechanical Screened Recycled Coarse Aggregates on Properties of Self-Compacting Concrete

**DOI:** 10.3390/ma16041483

**Published:** 2023-02-10

**Authors:** Waiching Tang, Mehrnoush Khavarian, Ali Yousefi, Bill Landenberger, Hongzhi Cui

**Affiliations:** 1 School of Architecture and Built Environment, The University of Newcastle, Callaghan, NSW 2308, Australia; 2 School of Environmental and Life Sciences, The University of Newcastle, Callaghan, NSW 2308, Australia; 3 College of Civil and Transportation Engineering, Shenzhen University, Shenzhen 518061, China

**Keywords:** recycled coarse aggregate, mechanical screening, self-compacting concrete, mechanical properties, microstructure

## Abstract

The use of recycled coarse aggregates (RA) in concrete is a sustainable alternative to non-renewable natural aggregate (NA) to fabricate concrete products using in concrete structures. However, the adhered mortar on the surface of RA would considerably impact the qualities of concrete products. As a practical treatment procedure, mechanical screening can remove the adhered mortar. This research aims to study the influence of mechanical screening on the fundamental properties of RA and the resulting self-compacting concrete (SCC). The RA were mechanically screened up to four times, and their physical properties including particle size distribution, water absorption, and crushing value were investigated. The properties of RA-SCC including workability, density, compressive and tensile strengths, modulus of elasticity, and microstructure were also examined. The results demonstrated that screening reduced the water absorption of RA from 6.26% to 5.33% and consequently enhanced the workability of RA-SCC. Furthermore, it was shown that increasing the screening up to twice improved the mechanical properties of concrete. In particular, screening increased the compressive strength of concrete by 15–35% compared to the concrete with unscreened RA. Similar improvements were found in tensile strength as well as the elastic modulus results. The microstructure of screened RA-SCC was comparable to that of the control concrete, showing minimal porosity and cracks along the interfacial transition zone. In conclusion, once or twice screening is recommended to the recycling facility plant to remove adequate amount of adhered mortar and fines while preventing damages to the RA. Improving the quality of RA via mechanical screening is one of the promising approaches to increase their potential for use in concrete, thereby reducing extraction of natural resources and promoting a circular economy.

## 1. Introduction

Concrete consumption is predicted to rise dramatically due to increasing investments in infrastructure projects around the world, reaching more than 10 billion tonnes per year [1]. It encompasses a wide range of transportation-related infrastructure projects such as roads and bridges as well as airports, railway lines, and waterways and canal systems. However, concrete production consumes large amounts of natural resources, and the construction industry is aware of the environmental impact of concrete production, creating the need to develop greener concrete. This has led to the development of several recycled concrete solutions that have the potential to have an impact on the future of the construction industry.

The global aggregates market size is expected to grow at a compound annual growth rate of 3.3% from 2020 to 2027. The expanding demand for aggregates used in concrete manufacturing has hugely impacted the natural resources and the environment [2]. To avoid resource depletion and promote sustainable development, finding an alternate source for natural aggregates (NA) is critical. According to Cement Concrete and Aggregates Australia [3], more than 162 million tonnes of aggregate per year, with demand expected to rise to 210 million tonnes by 2056 in Australia.

Furthermore, the rate of construction and demolition (C&D) waste to landfills has been steadily increasing, resulting in serious environmental consequences. According to the Department of Environment and Energy report [4], C&D waste accounts for 43% (20.4 Mt) of Australia’s total core waste, with concrete waste accounting for 81%. Currently, Australia’s C&D waste recycling rate is only 58%. However, several nations such as Japan, Denmark, and Estonia, have reached recycling rates of up to 98% [5]. This highlights the critical necessity to address the growing problem of concrete waste to achieve long-term growth. Other waste materials such as waste lathe scraps [6], waste tire [7] etc. have been successfully reutilised in concrete. In this regard, transforming the C&D waste into recycled aggregates (RA) to be utilised in concrete would not only reduce waste products in the construction industry but also certainly provide a noticeable reduction in the use of natural resources and carbon footprint in the construction industry [8].

Up to now, RA is primarily used to fabricate concrete products for non-structural applications as there are some concerns about their inferior properties [9]. Various parameters such as mix proportion, level of RA replacement, aggregates size, crushing method, and additive materials have been found to substantially impact the mechanical and hardened properties of concrete. However, concrete products containing RA have generally demonstrated lower mechanical properties than conventional concrete [10,11,12,13]. Indeed, RA has higher water absorption than NA, associated with the additional water adjustment to reach the required concrete workability, particularly for self-compacting concrete (SCC) [14,15,16,17,18,19]. These characteristics are mainly due to the old cement mortar that is weakly attached to the original NA. Therefore, removing the adhered mortar from the RA surface or strengthening the RA structure to make the aggregates usable and safe for structural purposes is necessary.

In the last decades, various methods such as ball milling [20,21], grinding [22,23], ultrasonic cleaning [24], acid treatment [25,26,27], coating and impregnation [28,29,30,31,32,33,34], carbonation [35], freeze-thaw method [36], conventional heating [37] and microwave process [38] have been proposed to strengthen the RA structure or remove the adhered mortar from the surface of RA. Indeed, removing the attached mortar from the surface of RA can improve the water absorption of RA and the interfacial bonding between RA and fresh cement paste. Dilbas et al. [20] and Savva et al. [22] demonstrated that mechanical grinding and ball milling can be efficient to remove weak adhered mortar, and resulting in lower water absorption and higher density of RA. Li et al. [39] suggested that mechanical grinding is far better than other types of treatments because of the simple procedure for producing high-quality RCA [39]. However, it can degrade the aggregates and cause microcracks in the RA structure [22,40]. Concrete waste is often ground and broken down to smaller sizes using a concrete crusher. Next, they are processed in the screening machine to separate RA with the preferable sizes. Etxeberria et al. [41] and Behera et al. [42] reported that crushers in the secondary step significantly improved the quality of aggregates by decreasing the content of adhered mortars. Tateyashiki et al. [43] proposed a two-step method of heating and grinding to produce high-quality RA. Ma et al. [44] adopted the heating process to decompose the limestone at 750 °C and separate aggregates from the cement paste. Another proposed method is ultrasonic water cleaning, which is suitable for removing loose and weak adhered mortars. However, it is ineffective to remove the strong cement adhered [40]. The study showed that the ultrasonic cleaning method could increase the 28-day compressive strength of concrete using RA by about 7% [24]. The hydration products of cement in the hardened paste can be dissolved in an acid solution. Thus, the acidic solution can be used to remove the adhered mortar effectively and enhance the quality of RA. Tam et al. [25] soaked RA in different acid solutions with concentration of 0.1 mole, including hydrochloric acid (HCl), sulphuric acid (H_2_SO_4_) and phosphoric acid (H_3_PO_4_). They found that HCl is the most effective one to remove the adhered mortar. However, the pre-soaking of RA in acidic solution leads to the formation of disposal wastes and the high amount of cement paste (alkaline phase) required to remove through a neutralization process. Acid treatment would also raise the cost of concrete manufacture. Mechanical screening is the most cost-effective and environmentally friendly of the suggested options for the building sector. Mechanical methods, on the other hand, usually result in the generation of finer aggregates. In comparison to thermal or chemical techniques, the screening procedure uses less energy [45]. In the current state, little attention has been devoted to mechanical treatment particularly mechanical screening and the number of screenings on the quality of RA and their incorporation in concrete.

The impact of the screening process on the fundamental characteristics of RA was investigated in this study. The physical properties of RA were also investigated, including particle size distribution, water absorption, and crushing value. Furthermore, the physical and mechanical properties of RA-containing self-compacting concrete (SCC) were examined. The effect of screening times and replacement levels (partial and full replacement) on properties of RA and RA-SCC were revealed. Scanning electron microscopy (SEM) was also used to investigate the microstructure and ITZ between cement paste and RA in SCC mixes. The efficiency of mechanical screening in removing adherent mortar and particles from the RA is demonstrated in this study and the optimal screening times could be recommended for practical application. Improving the quality of RA via mechanical screening could be one of the promising approaches to increase their potential for use in structural concrete, thereby reducing extraction of natural resources and promoting a circular economy.

## 2. Materials and Methods

### 2.1. Materials and Sample Preparation

**Figure 1:**
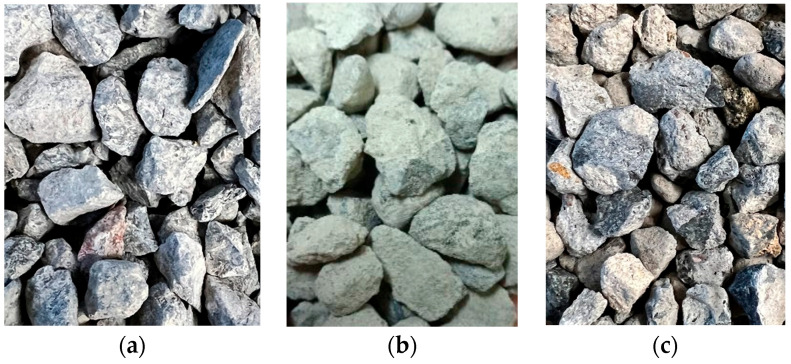
Coase aggregates (**a**) NA; (**b**) unscreened RAS0; (**c**) screened RAS4.

For this research, a general-purpose Portland cement (Type II) according to AS 3972 and fly ash in accordance with AS 3582.1 were used as cementitious materials. The chemical composition of the cement and fly ash are shown in Table 1. Crushed gravel with 10 mm was used as NA. RA with a size of 10 mm supplied by Concrush Pty Ltd., Teralba, NSW, Australia, were from a single source and screened up to four times. Figure 1 shows the coarse aggregates utilized in this study. The screening was performed using Powerscreen^®^ Chieftain 1400 (manufactured by Powerscreen, County Tyrone, Northern Ireland, UK) with a screen angle of 25° and each screen took an average of 8 min to go through. A total of 4500 kg of RA was screened, and the percentage of mass loss during the screening process is presented in Table 2. RAS0 refers to unscreened RA, whereas RAS1/2/3/4 refer to the number of screening applied. As listed in Table 2, there was a significant mass loss after every screening because of the removal of adhered mortar from the surface of RA. After screening, the residual fines were suitable as road base compaction material or a sub-base under concrete.

The NA and RA were immersed in water for 24 h and left for 1 h at room temperature before mixing to reach the dry surface condition. The ratio of water/cementitious material (cement and fly ash) was kept constant at 0.4. To obtain higher workability and achieve the SCC state, 100 mL of superplasticizers (Sikament NN supplied by Sika Australia Pty Limited, Wetherill Park, NSW, Australia) were added to the mixes. The mix details for 1 m^3^ concrete are listed in Table 3. According to Table 3, a control sample (C.S) with a 28-day compressive strength of 40 MPa and ten mixes containing RA with different screening times (RAS0, RAS1, RAS2, RAS3 and RAS4) were prepared. The RA mixes were intended to be used for structural applications.

Moreover, two series of RA-SCC samples with 50% and 100% of RA were fabricated to study the effects of RA replacement levels. In the mixing code, 50RA and 100RA referred to the mixes with 50% and 100% RA replacement, respectively, and the acronym S0–S4 referred to the RA with the different number of screenings. For instance, 100RAS3 is representative of SCC mixes with 100% replacement of RAS3 (RA, which screened three times).

### 2.2. 3D Model

**Figure 2:**
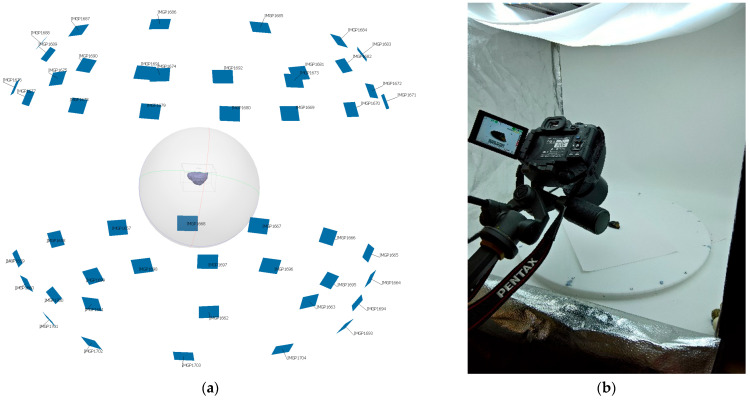
(**a**) Screen capture from AGI Soft Metashape, (**b**) turntable setup.

A 3D model was constructed from random RA samples after each screening stage to qualitatively reveal the effectiveness of screening. The models were built from photographs (typically 48) taken with a 24-megapixel digital SLR camera (ISO100, F32, 0.5-s shutter speed). The samples were mounted on a manual turntable inside a light tent to obtain multiple angles in diffuse lighting. Raw images were processed with AGISoft Metashape (version 1.55) to generate the 3D mesh and textures. Mesh detail varies from 300,000 faces to over 2,000,000 depending on the surface complexity and size of the sample. Figure 2 illustrates the screen capture from AGISoft Metashape. The mesh surface of the model is in the centre of the image in purple colour, and blue rectangles represent focal plane positions.

### 2.3. Physical Characteristic Testing of Aggregates

The main physical properties of aggregates (NA and RA), including particle size distribution, crushing value, and water absorption, were measured according to AS1141.11.1, AS 1141.21 and AS1141.6.1, respectively.

### 2.4. Fresh and Hardened Properties of RA-SCC

**Figure 3:**
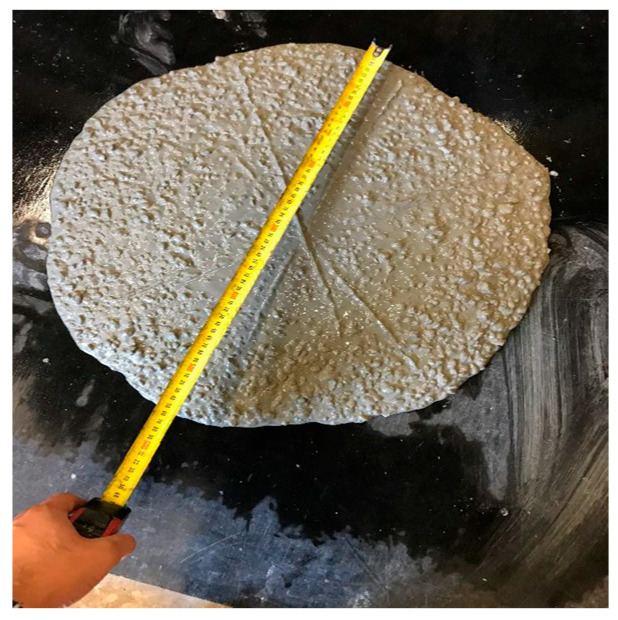
Measuring the slump flow diameter.

The fresh and hardened properties of RA-SCC, including workability, density, compressive and tensile strength, drying shrinkage and elastic modulus, were investigated. The workability of the SCC mixes was determined by the slump flow test according to AS1012.3.5. The density of hardened SCC was measured as per AS1012.12. Figure 3 illustrates the slump flow test conducted for the SCC samples. Workability is a critical property for SCC products, which is greatly affected by the water absorption of aggregates.

Cylindrical specimens with the dimension of Ø100 mm × 200 mm were cast to measure the compressive strength of the samples. All the SCC samples were cured and tested in correspondence with AS 1012.9. For the compressive strength, three specimens from each mix were tested at the ages of 7, 28, and 56 days. For the tensile test, three concrete cylinders (Ø100 mm × 200 mm) were prepared, and all the samples were cured and tested at the age of 28 days in correspondence with AS 1012.10. Moreover, the elastic modulus test was conducted on three cylinders (Ø100 mm × 200 mm) at 28 days as per AS 1012.17.

To study the microstructure of ITZ, scanning electron microscopy (SEM) analysis was conducted on the specimens. The samples were dried in an oven at 105 °C and treated before testing at the SEM analysis machine (ZEISS Sigma VP field emission scanning electron microscope, FE-SEM, manufactured by ZEISS, Jena, Germany) using a secondary electron detector.

## 3. Results

### 3.1. Characteristics of RA

#### 3.1.1. Particle Size Distribution

**Figure 4:**
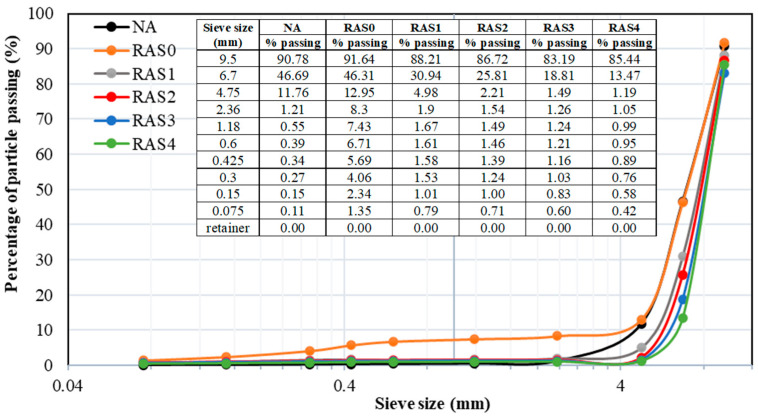
The particle size distribution of NA and RA with the different numbers of screening.

The effect of screening on the particle size distribution of RA was investigated and compared with NA. In general, the proper size distribution reduces the voids in the concrete matrix and decreases cement consumption. Therefore, well-graded RA would enhance the strength properties and durability of SCC. The particle size distribution graphs of NA and unscreened and screened RA are shown in Figure 4. There was a distinct grading trend for unscreened and screened RA. The size distribution of the RA with varying numbers of screenings was remarkably comparable. The graph also reflected that nearly half of the NA and unscreened RA samples could pass through a 6.7 mm sieve, whereas only 30% of the screened RA (RAS1) sample could, and the proportion declined as the number of screenings increased. According to the particle size distribution curves, the unscreened RA has a greater percentage of fine particles, which would result in higher water absorption and, as a result, lower workability [46].

#### 3.1.2. Aggregate Crushing Value

**Figure 5:**
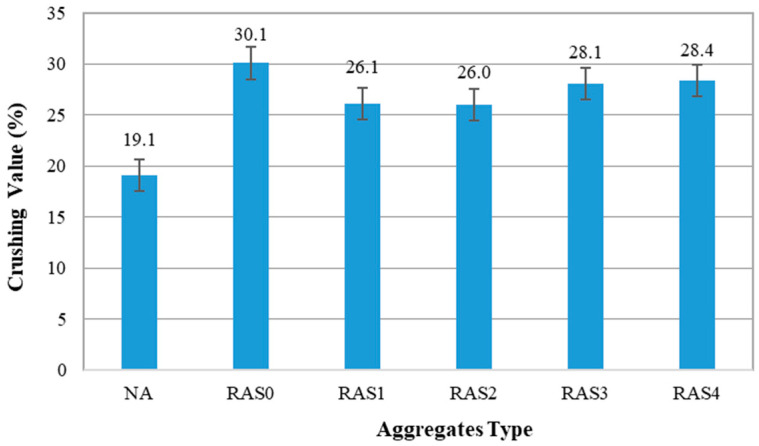
Crushing value of NA and RA with different numbers of screening.

Crushing value is a crucial metric for aggregate strength and, as a consequence, concrete mechanical performance. Figure 5 shows that the average aggregates crushing value outcomes for NA and RA with varying numbers of screenings. The crushing values obtained for RA samples were higher than for NA samples, indicating that RA had lower strength and toughness than NA. On the other hand, crushing values of screened RA samples are well within AS 1141.21’s standard limits (20–30%). The crushing value of NA was 19.1%, while it was 30.1% for RAS0 (unscreened RA). Screened RAs, on the other hand, had lower crushing values than unscreened RAs and were closer to the NA. The crushing values for RAS1, RAS2, RAS3 and RAS4 were 26.1%, 26.0%, 28.1% and 28.4%, respectively. The results reveal that once or twice screening would provide the best crushing value. The crushing values of RAS1 and RAS2 increased by up to 25% in comparison with RAS0. This implies that the strength and toughness of the RA can be increased after once or twice screening by removing adherent mortar from the surface of RA. Similar crushing value improvements can be found when recycled aggregates were mechanochemically treated [47]. The crushing value of 28.1% and 28.4% were obtained for RAS3 and RAS4, respectively. As the number of screening increases, the resistance to crushing decreases. It is possible that some of the RAs were rather damaged during the screening process due to excessive vibration. Hence, the number of screenings should be optimised to avoid any damage or loss of mechanical properties of RA.

#### 3.1.3. Water Absorption

**Figure 6:**
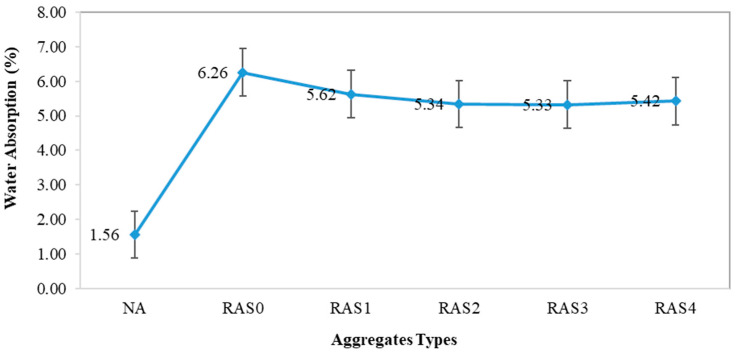
Water absorption results.

The water absorption of all samples, including NA and RA with the different screening numbers, is shown in Figure 6. The water absorption of NA was 1.56%. As expected, a higher water absorption obtained for RA with the different screening numbers ranged from 5.33% to 6.26%. The high water absorption of RA is because of the surface characteristics of RA and the attached old cement mortar paste [33]. As all the screened RA samples showed a higher water absorption value than the NA sample, the incorporation of RA in a saturated surface dry (SSD) state is proposed for the SCC production that also suggested by Saravanakumar et al. [27]. The results showed that one-time screening reduced the water absorption of the aggregates by 10.2% when compared to the unscreened aggregates (RAS0). Removing the associated mortar from the RA’s surface causes the water absorption value to drop after screening. The screening up to twice showed improvement in the water absorption results. However, further screening (three and four times) did not significantly reduce the water absorption of the aggregates. Similar to the findings reported by previous studies [20,21,22], the unscreened RA has the lowest density, maximum porosity, and highest water absorption than NA and screened RA, owing to more adhered mortar.

#### 3.1.4. The Surface Structure of RAs

**Figure 7:**
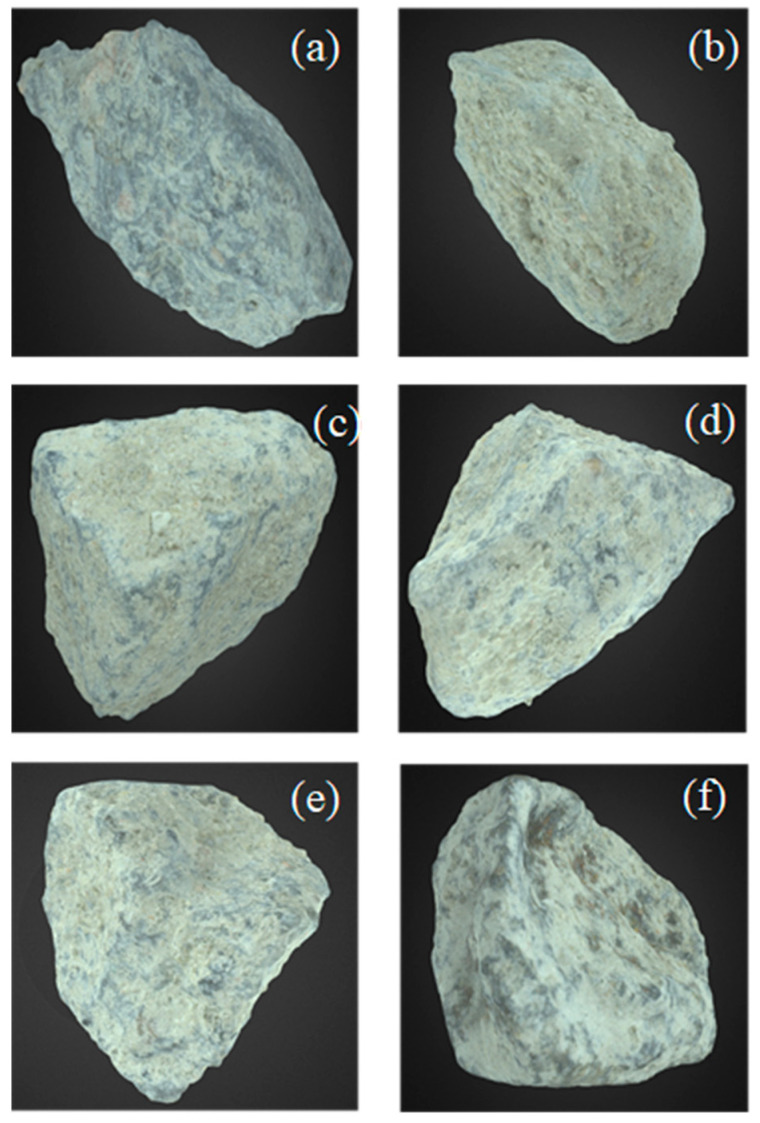
3D model observation of (**a**) NA, (**b**) RAS0, (**c**) RAS1, (**d**) RAS2, (**e**) RAS3 and (**f**) RAS4.

The surface structure of different aggregates, including NA, unscreened, and screened RA, was studied by 3D model images. Figure 7a–f illustrates the surface structure of NA, RAS0, RAS1, RAS2, RAS3 and RAS4, respectively. The screening process is designed to remove as much adhering cement mortar from RA as feasible. Figure 7 clearly illustrates that the RA with four times screening (RAS4) and unscreened RA (RAS0) had the largest and lowest amounts of adhered cement removal, respectively. It is evident that the screening procedure can efficiently remove the attached cement paste from the surface of RA.

### 3.2. Properties of RA-SCC

#### 3.2.1. Workability

The slump flow test was used to measure the workability and viscosity of SCC, which are essential features of fresh SCC [48]. The required time for the SCC mixes to attain a minimum diameter of 500 mm (T_500_), and the maximum flow diameter (D_max_) were measured during the slump flow test. The acceptable ranges for T_500_ and D_max_, according to EFNARC 2005 [49], are 2–7 s and 500–850 mm, respectively. Table 4 demonstrates D_max_ and T_500_ of all mixes. It is worth noting that the water absorption of RA is influenced by its porosity and permeability. The control mix (C.S) had the largest spread (D_max_ = 775 mm) and the quickest spread time (T_500_ = 2.08 s). Among the RA-containing mixes, 50RAS4 displayed the quickest spread time (T_500_ = 2.00 s) and the largest spread diameter with a D_max_ of 725 mm. Moreover, it was observed that as the number of screenings increased, T_500_ reduced, and D_max_ increased in RA-SCC samples with partial RA replacement. Based on the T_500_ results, it seems that screening would increase the viscosity of the RA mixes. It is also worth noting that the T_500_ value for 50RAS4 is lower than that of the control. More studies should be carried to confirm the findings. Nevertheless, the 100RAS0 had the lowest D_max_ and the longest slump flow time (T_500_) among all mixtures.

Indeed, the higher content of old cement mortar would absorb higher amount of water that results in lower workability and slower slump flow. It is noteworthy that the obtained slump flow times of all SCC mixes fell within the standard range of 2–7 s. The slump of 100RAS0 was 20% lower than the C.S mix, which agrees with the report of Santos et al. [50]. Due to the removal of the most part of old cement mortar in RA, mixes with screened RA generally showed higher slump flow values compared to the unscreened RA mix, which is in agreement with most of the related studies [20,51]. It can be inferred that the screening technique increased the workability of the SCC. Moreover, the average maximum flow diameter (D_max_) increased with the number of screenings, particularly in partial RA replacement mixtures.

#### 3.2.2. Density

**Figure 8:**
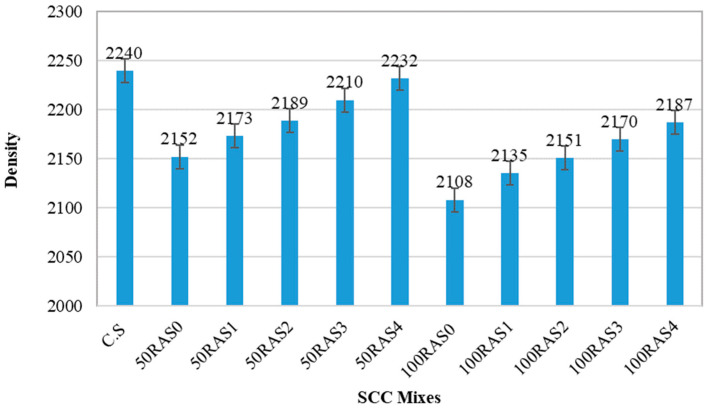
The average density of SCC mixes.

The average density of all SCC mixes, including control mix and mixes with unscreened and screened RA, is shown in Figure 8. Generally, the RA-SCC mixes had a lower density than the control sample (C.S) due to the decreased density of RA compared to NA. The mix with unscreened RA (100RAS0) had the lowest density of all the samples. Since RA is less dense than NA, the density of the SCC mixes dropped as the RA replacement percentage increased. With full or partial RA replacement, however, screening treatment increased the density of RA-SCC mixes. The density variation of 50RA mixes with partial replacement of RA with various screening numbers was 3.9%, 2.9%, 2.2%, 1.3% and 0.4% compared to C.S. Among all screened RA, the 50RAS4 mix sample had the lowest density variation with a value of 0.4%. The reason for this is that by increasing the number of screenings in SCC mixes, the density would increase due to the elimination of old and porous mortar. Similar to mechanically treated method [24], screening method would enhance the overall microstructural and density characteristics of the matrix.

#### 3.2.3. Compressive Strength

**Figure 9:**
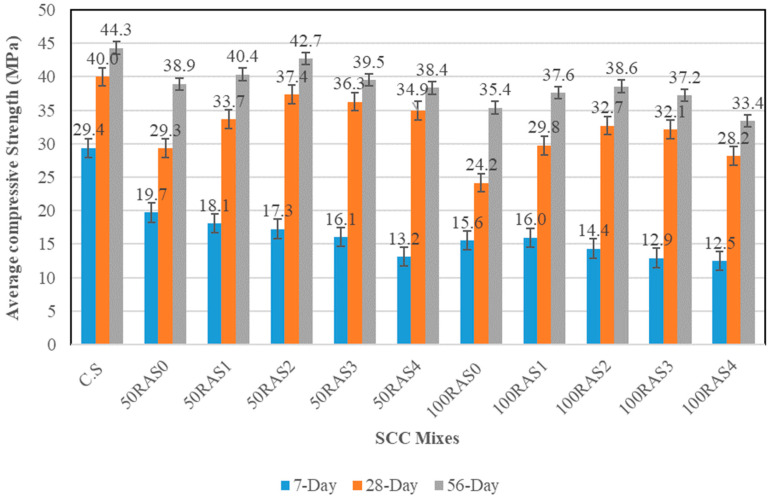
Average compressive strength results of SCC mixes.

The RA concrete samples failed in the same manner as that of control samples in compression. The compressive strengths of all designed SCC mixes at curing ages of 7, 28 and 56 days are displayed in Figure 9. The incorporation of RAs as a full or partial replacement in SCC mixes has a substantial impact on the compressive strength of SCC mixes at different ages. The compressive strength of concrete is affected by several factors, including mix proportion, size, quality and RA replacement ratio. As illustrated in Figure 9, the 28-day compressive values of RA-SCC samples are lower than that of the control sample (CS) by 7–40%, depending on the replacement levels and screening times. The main reason for this is that the strength of RA is lower compared to NA because of the higher crushing values as described before. In general, RA experienced crushing during the retrieval process leads to a weaker ITZ between cement and aggregates within the concrete, resulting in a lower compressive strength [52,53]. The 7-day compressive strength results exhibited a negligible difference among the RA-SCC samples. As illustrated in Figure 9, the 7-day compressive strength of SCC mixes incorporating full and partial replacement of unscreened and screened RA declined linearly with increasing screening times. The strength development of RA-SCC mixes was observed to be higher at a later age.

At the age of 28 days, the compressive strengths of SCC mixes with partial (50%) screened RA (50RAS1, 50RAS2, 50RAS3 and 50RAS4) were 15%, 27%, 24% and 19% greater than the unscreened RA mix sample (50RAS0), respectively. This implies that the screening procedure improved compressive strength. The 50RAS2 sample had the highest 28-day compressive strength among all the RA-SCC mixes, measuring 37.4 MPa. A considerable number of loose and weakly adhered RA mortars were eliminated during the screening phase, and their surface characteristics were enhanced, as shown in the 3D photos in Section 3.1.4. Furthermore, it was discovered that when the RA content of RA-SCC samples increased, the compressive strength of the samples dropped. This is consistent with previous findings, which showed that the compressive strength of SCC mixes reduced as the level of RA replacement increased [34]. At the age of 28 days, the improvement of compressive strength of 100RAS1, 100RAS2, 100RAS3 and 100RAS4 samples was about 23%, 35%, 33% and 17%, compared to the concrete with unscreened RA (100RAS0). This enhancement demonstrates that screening can effectively remove an adequate amount of attached mortar to improve the interfacial transition zone (ITZ) between the screened RA and fresh cement paste, enhancing compressive strength. More discussion has been made in the microstructure section. However, when the number of screenings applied is more than twice, the compressive strength of screened RA-SCC tends to drop as shown in Figure 9. At 56 days, the RA-SCC mixes containing unscreened and screened RA with full and partial replacement exhibited a similar trend to the compressive strengths at 28 days.

#### 3.2.4. Tensile Test

**Figure 10:**
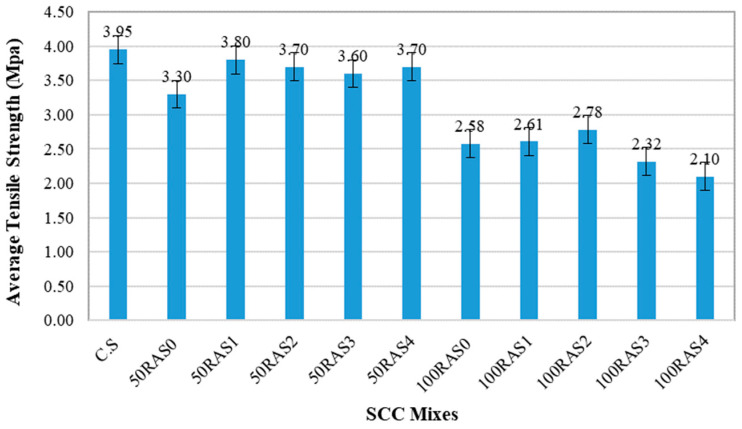
Tensile strength of SCC mixes at 28 days.

Figure 10 displays the tensile strength results of RA-SCC mixes at 28 days. As can be observed, the C.S sample displayed the highest tensile strength of nearly 4 MPa. The tensile strengths of samples with partial replacement of screened RA are generally higher than unscreened RA sample. In general, the tensile strength results follow a fairly similar trend to the compressive strength results. However, the effect of screening times on tensile strength is not significant. The tensile strengths of samples with full RA replacement are much lower than those with partial RA replacement. When the number of screenings applied was more than twice, the tensile properties tended to decline, similar to the compressive strength values. The SCC mixes of 100RAS0, 100RAS1 and 100RAS2 were roughly 35%, 34% and 30% lower than the C.S mix, respectively. It should be noticed that as the RA replacement levels increase, the number of air voids in the concrete matrix increase as well. This is the main reason the tensile strengths of samples with full replacement are lower than their counterparts with partial replacement. This is consistent with previous findings, which showed that the higher the RA replacement level, the lower the tensile strength. [34,54]. Based on the strength test results, it seems that screening up to two times is recommended as the tensile strength of the 100RAS2 sample was the highest when compared to the unscreened and screened samples with full RA replacement.

#### 3.2.5. Elastic Modulus

**Figure 11:**
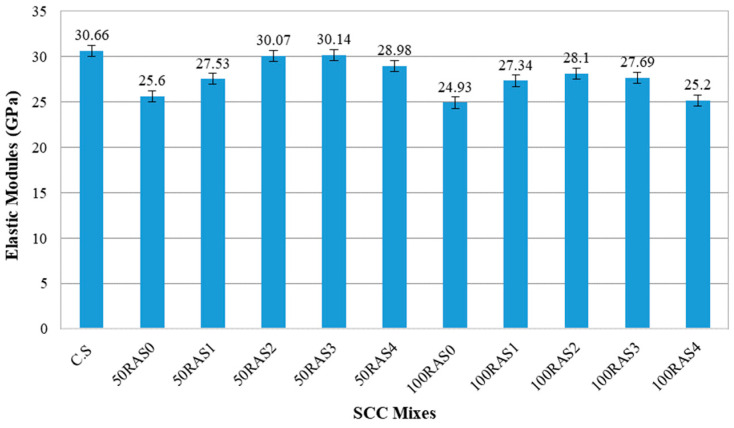
Elastic modules result of SCC mixes.

The stiffness of RA-SCC mixes was determined based on the modulus of elasticity. Several factors have been reported to have substantial contributions to the stiffness of concrete, including aggregate density and porosity, matrix, cement paste rigidity, and ITZ structure [55]. Figure 11 illustrates the elastic modulus results of RA-SCC mixes compared to the control (C.S mix). When compared to the control sample, all RA-SCC mixes had a lower elastic modulus. The main reason is due to the porous nature of RAs and their low concrete density as well as the weak relationship between old and new ITZs [14,15]. The elastic modulus of the RA-SCC mixtures with full RA replacement was lower than that of their partial replacement counterparts. The modulus of elasticity of RA-SCC decreases with the level of RA replacement, which is consistent with prior research results [12,13,56,57]. The elastic modulus of 50RAS2 is very close to that of C.S, and higher than those of 50RAS0 and 50RAS1 by 9% and 17%, respectively. The elastic modulus of 100RAS1 and 100RAS2 was around 10% and 13% higher than that of 100RAS0. Apparently, using mechanical screened RA can improve the elastic modulus behaviour of RA-SCC mixes. However, comparing between 100RAS2 and 100RAS4, a 10% drop in the elastic modulus observed when the screening increased from 2 to 4 times. Based on the mechanical test findings, it is clear that excessive screening can degrade RA and affect the strengths and elastic modulus, so it is advised that screening be performed no more than two times to gain the benefits of screening.

#### 3.2.6. Microstructure Observation

**Figure 12:**
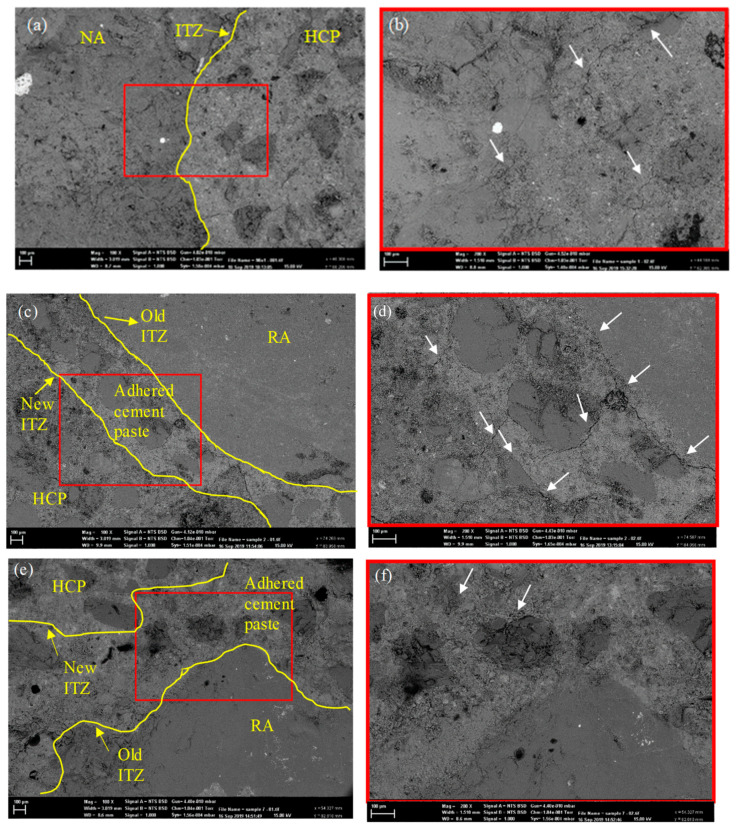

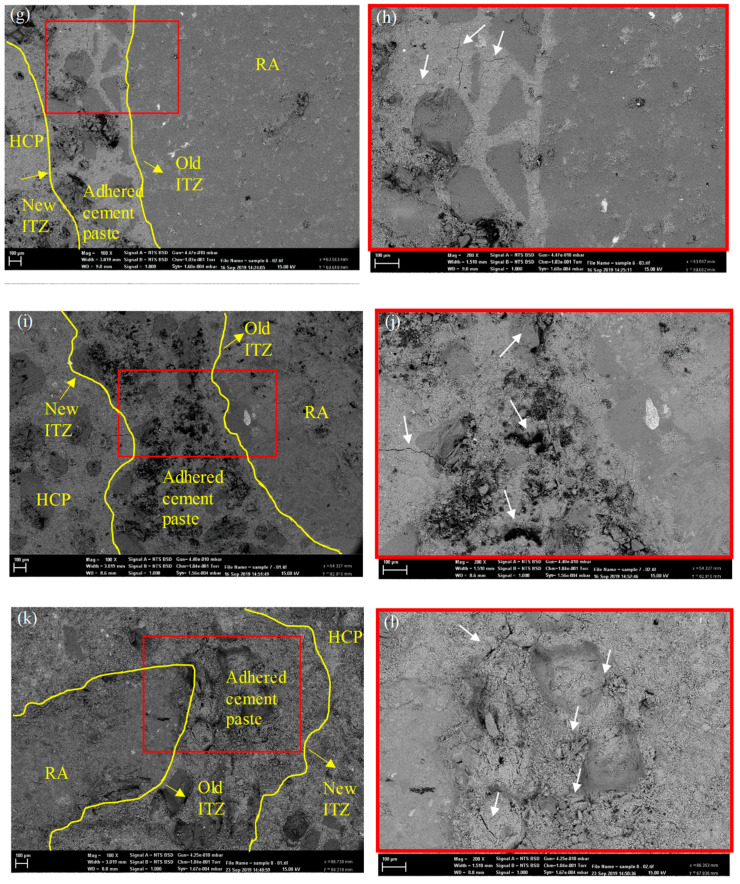
SEM images of (**a**,**b**) C.S, (**c**,**d**) 100RAS0, (**e**,**f**) 100RAS1 (**g**,**h**) 100RAS2, (**i**,**j**) 100RAS3 and (**k**,**l**) 100RAS4 mixes (in different magnifications).

The microstructure and interfacial transition zones (ITZs) in the RA-SCC samples were investigated using SEM. The SEM images of SCC mixes with different magnifications are shown in Figure 12a–j. Figure 12a,b depicts the SEM image of the C.S sample, including the ITZ between the NA and the hardened cement paste (shown by the yellow line). It demonstrates a strong bonding between NA and hardened cement paste with minimal pores and voids. Figure 12c,d demonstrates the SEM images of 100RAS0 (SCC sample made of unscreened RA). As expected, the microstructure of the 100RAS0 sample differs from that of C.S. The yellow lines depict the old ITZs (between the NA and cement paste) and the new ITZs (between the RA and cement paste). The long and extensive microcracks found throughout the old ITZ indicate a sloppy and weak connection between the cement mortar and the RA. The micro-cracks are depicted by white arrows in Figure 12. Micro-cracks, initiate and expand in the ITZ, are considered the weakest part of the cement matrix [58]. As observed in the compression test results (Figure 9), the compressive strength of 100RAS0 dropped compared to the C.S sample. This agrees with prior research studies that the old ITZ would impact the microstructure and strength performances [59,60,61], especially with increased replacement of RA results in a large amount of porosity [62]. Based on the SEM images, it can be determined that unscreened RA has a lower density, higher water absorption, and inferior mechanical qualities due to a large amount of adhered old cement mortar and the presence of micro-cracks throughout the attached old cement.

The SEM images of the 100RAS1, 100RAS2, 100RAS3, and 100RAS4 samples are shown in Figure 12e–l. As indicated by the arrows, minimal micro-cracks were found in the microstructure and ITZs of 100RAS1 and 100RAS2 samples when compared to the unscreened sample. In the 100RAS1 and 100RAS2 samples, the microcracks are predominantly single, with very little networking. The crack development through the attached old cement mortar is visible in the SEM images of the 100RAS3 (Figure 12i,j). However, the detected cracks were mostly single, with only a few cracks networking.

More microcracks can be seen on the adhered cement mortar of the 100RAS4 sample in Figure 12k,l. The existence of micro-cracks and pores and inadequate physical and chemical bonding between RA and cement paste indicate faults in the microstructure of RA, which decrease the mechanical capabilities [63]. The growth of microcracks in the 100RAS3 and 100RAS4 mixtures is a major contributor in lowering their physical and mechanical qualities, as proved in the previous sections. It can be revealed that increasing the screening time generates some microcracks along the old ITZs. Still, it does not significantly affect the SCC microstructure. In general, screening up to 2 times can improve the microstructure of ITZ.

## 4. Conclusions

This study investigated the effect of screening times and replacement levels (partial and full replacement) on the physical and mechanical properties of RA and RA-SCC mixes. Scanning electron microscopy (SEM) was also used to investigate the microstructure and ITZ between cement paste and RA in SCC mixes. The efficiency of mechanical screening in removing adherent mortar and particles from the RA is demonstrated in this study. The following conclusions can be drawn based on the findings.

1. The size distribution results revealed that unscreened RA contains a large number of fine aggregates with high porosity and consequently a higher water absorption.
2. Screening improved the crushing values of RA, and all of the screened RA lie well within standard limits (20–30%) following AS 1141.21.
3. The water absorption of RA decreased as the number of screening increased due to the removal of a greater amount of adhered mortar from the surface of RA. The water absorption of RA reduced from 6.12% to 5.34% after screening two times.
4. SCC mixes with screened RA had higher maximum flow diameters (D_max_) than those containing unscreened RA. The slump flow times of all SCC mixes fell within the standard range of 2–7 s.
5. Screening increased the 28-day compressive strength of SCC mixes by 15–28% on average, compared to the mixes with unscreened RA. The results of tensile strength and elastic modulus show similar patterns. However, increasing the screening more than twice might increase micro-cracks along the old ITZs, affecting the mechanical properties.
6. The microstructure of concrete samples containing screened RAs was generally comparable to that of the control concrete, with minimal pores, voids and cracks along the interfacial transition zones.
7. Based on mechanical performance, 50RA mixes with RA screened either once or twice performed the best among the RA mixes. Therefore, once or twice screening is recommended to the recycling facility plant to remove the adequate amount of adhered mortar and reduce fines from the RA.
8. The mechanical screening is a practical approach to improve the quality of RA and the final concrete, owing to its contribution to removing adhered mortar on the surface of RA. However, the environmental impacts of screening need to be examined further, as each incurs expenses, takes time, and consumes energy. Nevertheless, the residual fines after screening are appropriate for use as a road base compaction material or a sub-base under concrete, which could offset their environmental consequences.

## Figures and Tables

**Table 1:** Chemical Composition of Cement and Fly Ash (wt%).

Type	Na_2_O	MgO	Al_2_O_3_	CaO	TiO_2_	SiO_2_	Fe_2_O_3_
Fly Ash	0.49	0.42	24.00	1.59	0.92	65.90	2.87
Cement	0.20	1.20	5.90	65.85	0.54	21.14	3.10

**Table 2:** Screening information.

RA Type	Times of Screening	Mass Loss (%)
RAS0	0	0
RAS1	1	7.8
RAS2	2	13.3
RAS3	3	16.7
RAS4	4	17.8

**Table 3:** The details of mix proportion for 1 m^3^ concrete.

Mixing Code	NA (kg)	RA (kg)	Sand (kg)	Cement (kg)	Fly Ash (kg)	Water (L)	S.P (mL)
C.S	870	-	645	320	220	216	100
50RAS0	435	435	645	320	220	216	100
50RAS1	435	435	645	320	220	216	100
50RAS2	435	435	645	320	220	216	100
50RAS3	435	435	645	320	220	216	100
50RAS4	435	435	645	320	220	216	100
100RAS0	-	870	645	320	220	216	100
100RAS1	-	870	645	320	220	216	100
100RAS2	-	870	645	320	220	216	100
100RAS3	-	870	645	320	220	216	100
100RAS4	-	870	645	320	220	216	100

**Table 4:** The results of the Slump flow test.

Mixing Code	T_500_ (s)	Average Diameter (mm)
C.S	2.08	775.0
50RAS0	2.73	592.5
50RAS1	2.46	602.5
50RAS2	2.33	662.5
50RAS3	2.20	705.0
50RAS4	2.00	725.0
100RAS0	3.79	585.0
100RAS1	2.01	665.0
100RAS2	2.05	656.0
100RAS3	2.43	751.0
100RAS4	2.72	658.0

## Data Availability

Not applicable.
